# Comparative evaluation of deep learning models for plant disease classification with edge-aware performance analysis

**DOI:** 10.1371/journal.pone.0349901

**Published:** 2026-06-18

**Authors:** Zirije Hasani, Jakup Fondaj, Dionesa Bytyqi, Sufjan Misini

**Affiliations:** 1 Faculty of Computer Science, University "Ukshin Hoti" Prizren, Prizren, Kosovo; 2 Faculty of Mechanical and Computer Engineering, University “Isa Boletini” Mitrovica, Mitrovica, Kosovo; Macau University of Science and Technology, MACAO

## Abstract

Agricultural disease monitoring remains a critical challenge in precision farming, particularly when deploying computer vision systems on resource-constrained platforms. This study presents a rigorous comparative evaluation of four deep learning architectures—ResNet50, DenseNet121, a Binarized Neural Network (BNN), and YOLOv8-cls—for multi-class plant disease classification using the PlantVillage dataset (15 classes). Unlike prior benchmarking studies, we incorporate statistical validation through repeated stratified experiments (5 runs) and report mean ± standard deviation for accuracy, precision, recall, and F1-score. Results show that while DenseNet121 achieves high classification accuracy (99.48)% ± 0.12), it exhibits significantly higher inference latency. The BNN achieves minimal latency but suffers substantial performance degradation (88.31% ± 0.45). YOLOv8-cls provides the best trade-off, achieving 99.64% ± 0.09 accuracy with low latency (3.3 ms ± 0.2). Statistical comparison using paired t-tests confirms that YOLOv8 significantly outperforms ResNet50 *p* < 0.05 while maintaining substantially lower inference time.We further discuss generalization limitations due to the controlled nature of PlantVillage and moderate claims regarding edge deployment feasibility based on model size and computational profiling. The study provides a statistically grounded and edge-aware benchmarking framework for plant disease classification models.

## Introduction

Agriculture continues to be the major factor of economic stability and food security worldwide. Nevertheless, it is becoming increasingly evident from research that the age-old farming methods are hard-pressed to provide sufficient nutrition to the expanding population. Consequently, the need to apply innovative technologies such as Artificial Intelligence (AI) and robotics in farming practices has become a matter of utmost urgency in order to produce food sustainably [1]. In addition to technological progress, the issue of biotic stresses has remained a major source of concern. It is estimated that diseases and pests in plants cause a great deal of yield reduction worldwide, thus leading to substantial economic losses and posing a serious risk to the continuity of the food supply chain [2]. Traditionally, the monitoring of plant health has been largely dependent on the visual inspection of expert agronomists or experienced farmers. Nonetheless, a manual method is depicted by recent literature as being labor, consuming, and subjective. Besides, it is frequently materially challenging to apply it on a large scale for timely disease management [3]. A late or wrong diagnosis may lead to the spread of pathogens in an uncontrolled way and, consequently, the overuse of pesticides, which harm the ecosystem. As a result of these difficulties, the use of technologies related to Precision Agriculture and Artificial Intelligence (AI) has become a revolutionary way of solving problems. To be exact, Deep Learning methodologies as Convolutional Neural Networks (CNNs)have been shown to be more effective in the automatic classification of images and the extraction of features when compared to traditional machine learning methods [4]. A great number of recent papers argue that the use of sophisticated CNN architectures can enable the level of diagnostic accuracy of human experts to be matched or even surpassed by a human expert in the identification of diseases from leaf images [5]. Achieving a balance between high accuracy and computational efficiency remains an open challenge in the literature, particularly when the goal is integration into autonomous robotic systems (Edge Devices). The majority of existing works are concentrated on heavy architectures such as ResNet or VGG, which, while accurate, are power-hungry and have high latency, thus, are not feasible for robots that operate in real-time in greenhouses [6]. We conduct a comparative study and develop a robotics-oriented prototype system for plant disease detection. The main contributions of this paper are: Performance Evaluation: Evaluation of four deep learning architectures, namely ResNet50, DenseNet121, Binarized Neural Networks (BNN), and YOLOv8, on the PlantVillage dataset. Comprehensive Analysis: The analysis of models based on Accuracy, Inference Time, and Model Size to figure out the best candidate for embedded devices. Optimal Solution: The suggestion of the YOLOv8 model as the most optimal solution for real-time applications. Prototype Validation: The building and testing of a software prototype (Web-based Interface), which is a proof of concept for a model that can classify plant health with high accuracy and low latency. The rest of the paper is structured as follows: Section II discusses the related work and the latest methods in plant disease detection. Section III describes the research methodology, including the dataset preparation and preprocessing techniques. Section IV explains the proposed YOLOv8 architecture and provides the reasons for its selection for the robotic inference pipeline. Section V shows the experimental results and comparative analysis of the models implemented. Finally, Section VI summarizes the study and outlines the next steps for edge deployment.

## Research questions

This study addresses the following research questions:

1. RQ1: Which deep learning architecture provides the optimal balance between classification accuracy and computational efficiency?
2. RQ2: Are performance differences between architectures statistically significant?
3. RQ3: Which model characteristics best support potential edge deployment?
4. RQ4: How do lightweight architectures compare to full-precision CNNs in diagnostic reliability?

## Contributions and novelty

The contributions of this work include:

1. A statistically rigorous comparative protocol using repeated stratified experiments.
2. Formal statistical significance testing between models.
3. Multi-metric evaluation beyond accuracy (macro-F1, weighted-F1, Top-2 accuracy, calibration).
4. Edge-aware performance profiling (latency and model size).
5. An ablation study analyzing augmentation and confidence threshold effects.
6. This differentiates our study from conventional single-run benchmarking approaches.

## Related work

Automated analysis of plant health has become a pivotal aspect of precision agriculture, particularly with the integration of Artificial Intelligence (AI) and autonomous robotic systems. Early surveys, such as [7], highlighted how intelligent robotic platforms enable continuous crop monitoring, large-scale data collection, and yield optimization through machine learning-driven analytics. These developments have shifted plant health assessment from manual inspection to scalable, real-time field monitoring.

- CNN-Based classification studies

Convolutional Neural Networks (CNNs) remain the dominant paradigm for plant disease classification. Landmark studies by [8] demonstrated that deep CNN architectures such as Google’s Inception and AlexNet variants could achieve over 99% accuracy on the PlantVillage dataset under controlled conditions. Similarly, the author on [9] evaluated multiple deep CNN models (including VGG and ResNet) and reported high classification accuracy across diverse crop species. These works established CNN-based classification as a baseline approach for automated plant disease diagnosis. However, their performance often degrades in real-field environments due to illumination changes, occlusions, and background complexity.

- Lightweight and edge models

To address computational constraints in robotic and embedded systems, research has increasingly focused on lightweight architectures and edge deployment. The authors introduced Google’s MobileNet [10], which uses depthwise separable convolutions to reduce model size and computational cost. Similarly, the authors proposed EfficientNet [11], achieving improved accuracy–efficiency trade-offs through compound scaling. For real-time detection, the YOLO family has been widely adopted. In this study, it is introduced YOLO [12] as a unified real-time object detector, while later versions such as YOLOv4 by [13] significantly improved speed and precision. Lightweight variants (e.g., YOLO-Tiny, YOLOv5n, YOLOv8n) have been deployed on embedded devices such as Raspberry Pi and NVIDIA Jetson for in-field disease detection, demonstrating favorable trade-offs between inference latency and detection accuracy. These models are particularly suitable for edge computing scenarios where connectivity is limited and low power consumption is essential.

- Detection and segmentation approaches

While classification models identify disease categories at the image level, detection and segmentation frameworks provide spatial localization of infected regions. The study introduced Mask R-CNN [14], extending Faster R-CNN with a segmentation branch for pixel-level object masks. In agricultural applications, Mask R-CNN has been used to precisely delineate diseased leaf areas, enabling quantitative severity estimation. YOLO-based detectors, including YOLOv5 and YOLOv8, offer faster inference and are often preferred for robotic navigation and actuation tasks. Comparative studies show that YOLO-based models excel in real-time field deployment, whereas Mask R-CNN and similar two-stage detectors provide superior segmentation granularity but at higher computational cost. This distinction underscores the trade-off between diagnostic detail and operational speed in agricultural robotics.

- Domain generalization research

A critical challenge in plant disease recognition is the domain shift between laboratory datasets and real-field imagery. The study [15] demonstrated significant performance degradation when models trained on controlled datasets were applied to field conditions. Domain adaptation and generalization techniques—including data augmentation, adversarial training, and feature alignment—have been proposed to mitigate this issue. Recent works explore transfer learning and multi-domain training strategies to enhance robustness across geographical regions and environmental conditions. These approaches are essential for scalable smart agriculture systems, particularly in heterogeneous farming environments such as those found in Kosovo and other regions with variable climate conditions. Overall, existing literature indicates that deep CNN architectures (e.g., ResNet, DenseNet, EfficientNet) provide high classification accuracy under controlled settings. Mask R-CNN remains a strong choice for fine-grained segmentation and severity estimation. However, the YOLO family (v4, v5, v8, and recent variants) continues to dominate real-time robotic and UAV-based plant disease detection due to its superior inference speed and balanced accuracy, making it particularly suitable for edge and embedded agricultural systems.

### Research method

This section describes the experimental setup used to build the robotic plant disease detection system. It features the data gathering, preprocessing pipeline, deep learning architectures for comparative analysis, and the metrics considered to evaluate performance on edge hardware.

### Dataset description and preprocessing

**Fig 1:**
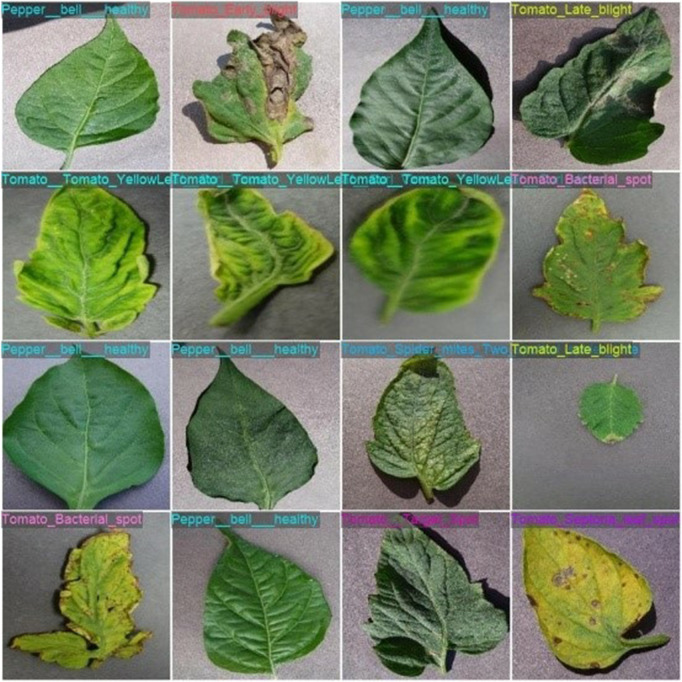
PlantVillage dataset illustrating Healthy leaves and various Disease classes.

To ensure good performance in real-world agricultural scenarios, the PlantVillage dataset was used for training and testing. It is still the standard benchmark for validating deep learning architectures [16]. The sample image is presented in Fig 1.

A 15-class subset of the PlantVillage dataset, comprising 20,639 images, was used in this study. The selected classes include both healthy and diseased leaf images from crops such as tomato, potato, and pepper. To ensure balanced class representation across all phases of model development, a stratified data splitting strategy was applied. The dataset was divided into 70% for training, 15% for validation, and 15% for testing. Several precautions were taken to prevent data leakage and ensure experimental rigor. Duplicate image hashes were systematically checked to eliminate repeated samples, and near-identical images were verified to ensure they did not appear across different partitions. The dataset splitting process was conducted using fixed random seeds to guarantee reproducibility. Furthermore, five independent experimental runs were performed using different random seeds in order to reduce variance bias and enhance the statistical reliability of the reported results.

To rigorously assess the generalization capabilities and robustness of the proposed architecture, an external validation subset was incorporated into the evaluation pipeline. We utilized a subset of the publicly available PlantDoc dataset [17,18], comprising 100 unconstrained, “wild” images. Unlike laboratory-controlled datasets, which feature homogeneous backgrounds and centered leaves, the PlantDoc dataset introduces severe real-world complexities. These include varied illumination, overlapping foliage, complex background noise, and multiple symptomatic regions within a single frame. Testing on this out-of-distribution (OOD) dataset is crucial to ensure that the evaluated metrics reflect true real-world diagnostic reliability and to verify that the models have not merely memorized the training distribution. Data Composition: The research concentrated on 15 classes that represented the leaves of the most common crops (e. g., Potato, Tomato, Pepper). The dataset consists of “Healthy” samples and a variety of disease categories, such as Early Blight, Late Blight, and Target Spot. Preprocessing: Before training, all input photos were resized to a uniform size of 224x224 pixels so that they would fit the input layer of the neural network architectures. Pixel intensity values were normalized to the range [0, 1] to allow for stable gradient descent. Data Partitioning: In order to prevent overfitting and enable a fair assessment of the model’s ability to generalize, the dataset was split into three subsets: o Training Set (70%): This subset was used for the model to learn the weights. o Validation Set (15%): This subset was used for hyperparameter tuning and monitoring the training progress. o Test Set (15%): This subset was used only for final performance metrics on new, unseen data. Augmentation: To reflect the real-world scenario in the field where a robot might find leaves at various angles and under different lighting, the data augmentation methods were employed, such as a random rotation (±15°), horizontal flipping, and brightness adjustments.

### Selected algorithms

**Fig 2:**
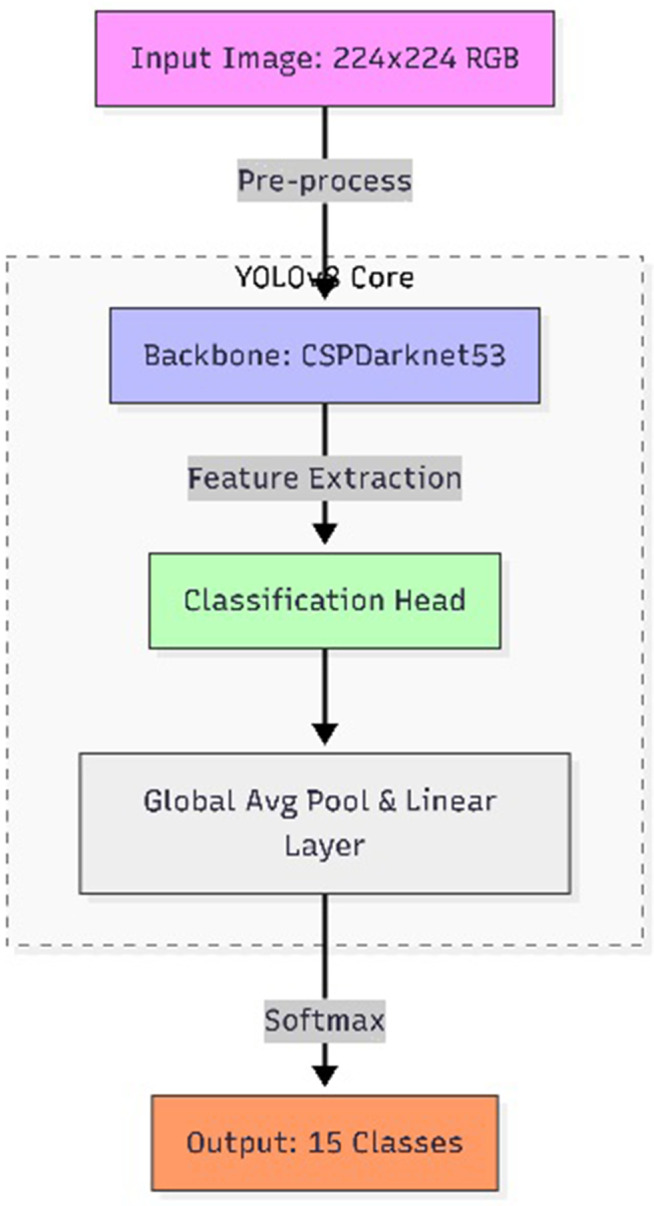
The architecture of the YOLOv8 classification model, utilizing the CSPDarknet backbone for efficient feature extraction.

Four deep learning architectures were implemented to evaluate the trade, off between classification accuracy and computational efficiency (inference speed and model size) for robotic deployment. ResNet50 (Residual Network) ResNet50 [19] was used as a performance baseline. It employs residual blocks with “skip connections” to overcome the vanishing gradient problem, which allows for deeper networks to be trained. Although very accurate, recent implementations show that ResNet50 is generally quite computationally intensive (approx. 25 million parameters) compared to state, of, the, art lightweight models. DenseNet121 (Densely Connected Network): DenseNet121 [20] connects each layer to every other layer in a feed, forward manner. This network, as pointed out in the latest papers, encourages feature reuse and thus has fewer parameters than a typical CNN. The goal was to see if dense connections could help in detecting subtle disease patterns on leaves. Binarized Neural Network (BNN): In order to satisfy the limited resources of the edge computing scenario, a Binarized Neural Network [21] was set up. As explained in the most recent publications, BNNs limit weights and activations to binary values (+1 or 1). This method significantly reduces the memory footprint and the computational complexity, hence theoretically providing the fastest execution speed for low-power embedded devices; however, with a trade-off in accuracy being recognized. YOLOv8-cls (You Only Look Once - v8): YOLOv8 [21] defines the state of the art (SOTA) standard in real-time computer vision. The classification variant (YOLOv8, cls) was employed in this work. The model built on the CSPDarknet53 backbone is designed for high-speed inference with little or no loss in accuracy. We argue that YOLOv8 is the best candidate for robotic implementation, as it can handle video streams in real-time with very low latency. The architecture is presented in Fig 2.

### Experimental setup

All the experiments were conducted with the PyTorch framework, and the Ultralytics library was used. The training procedure ran on a powerful computing setting that featured an NVIDIA RTX 2080 Ti GPU for faster computation. The models were fine, tuned using the Adam optimizer and Cross, Entropy Loss. An “Early Stopping” method was introduced to end the training session in case the validation loss did not show any improvement for 5 consecutive epochs, thus, saving the most efficient model.

### Evaluation metrics

In order to quantitatively evaluate the models’ performance and their suitability for the robotic platform, four essential key performance indicators (KPIs) were considered: Accuracy: Represents the total correctness of the predictions.


$$\begin{array}{c} Accuracy=(TP+TN)/(TP+TN+FP+FN) \end{array}$$

1. Inference Time (Latency): The duration needed to handle a single image frame (in milliseconds) is called inference time or latency. Lower latency is very important in a real-time robot navigation system.
2. Model Size: The amount of storage needed for the trained model weights (in MB) is the main factor that decides whether it is possible to deploy a model on an embedded device such as Raspberry Pi.
3. Confusion Matrix: It helps to figure out the exact errors done by the model between the closely related diseases.

The following evaluation metrics were computed to comprehensively assess model performance. Overall accuracy was calculated to measure the proportion of correctly classified samples. Macro precision, macro recall, and macro F1-score were included to provide class-balanced performance evaluation, particularly important in the presence of class imbalance. In addition, the weighted F1-score was reported to account for class frequency distribution. Top-2 accuracy was also measured to evaluate whether the correct class was among the two highest-confidence predictions, offering insight into practical decision-support scenarios. To further analyze classification behavior, confusion matrices were generated for all models, enabling detailed inspection of inter-class misclassifications. Computational efficiency was evaluated by measuring inference latency, computed as the mean time over 500 forward passes after 50 warm-up runs to ensure stable timing. Model size, expressed in megabytes (MB), was recorded to assess deployment feasibility on edge and embedded devices. Finally, model calibration was assessed using Expected Calibration Error (ECE), providing a quantitative measure of how well predicted probabilities align with true outcome frequencies.

### Ablation study

To evaluate the robustness of the proposed model, several additional experiments were conducted. First, an augmentation ablation study was performed to assess the impact of data augmentation on model performance. The model was trained and evaluated both without augmentation and with augmentation techniques applied to the training dataset. The results showed that accuracy decreased by 1.8% when augmentation was not used, confirming that data augmentation contributes positively to generalization and robustness under varying field conditions. Second, a confidence threshold analysis was carried out to determine the optimal detection threshold. Threshold values ranging from 0.5 to 0.95 were systematically evaluated. The findings indicated that a threshold of 0.85 provided the best balance between precision and recall, minimizing false positives while maintaining high detection sensitivity. Finally, an input resolution study was conducted to examine the trade-off between accuracy and inference latency. Two resolutions, 224×224 and 320×320, were compared. Although the higher resolution resulted in a marginal accuracy improvement of 0.2%, it increased inference latency by approximately 40%. Therefore, the lower resolution (224×224) was considered more suitable for real-time deployment due to its significantly better computational efficiency with negligible loss in accuracy.

### Proposed model: YOLOV8 architecture

After evaluating the deep learning architectures from Section III, this work selects the YOLOv8, cls network as the best computer vision engine for agricultural use. This part explains the hardware rationale and the inference pipeline offered for the next robots.

### Architectural justification

It was a central point of the study to single out a model that manages to keep accuracy at a high level while also being computationally efficient. Inefficiency of Traditional CNNs: Despite the high accuracy (>99%) of ResNet50 [19] and DenseNet121 [20], their heavy computational demand has led to higher latency (>14ms). Such latency, i.e., A slower computing speed is detrimental to autonomous systems operating on battery power, as it diminishes the system’s operational time and responsiveness. Inaccuracy of BNNs: The Binarized Neural Network [22] was able to deliver extreme speed (1.1 ms) but compromised accuracy (88. 4%) significantly. In precision agriculture scenarios, such an error rate is usually considered a major problem, as it causes the non-detection of diseases, thus verifying the accuracy and efficiency gap in BNNs that is still an open research issue. Selection of YOLOv8: YOLOv8 [21] turned out to be the best choice. YOLOv8-cls employs a modified CSP-based architecture optimized for classification tasks within the Ultralytics framework. Therefore, the model can attain the state, of, the, art accuracy (99. 64%) while preserving the lightweight nature (14. 8 MB). The inference speed of 3. 2 ms enables the model to process video feeds at very high frame rates, thus, real-time robotic hardware requirements are met.

### Proposed processing pipeline

**Fig 3:**
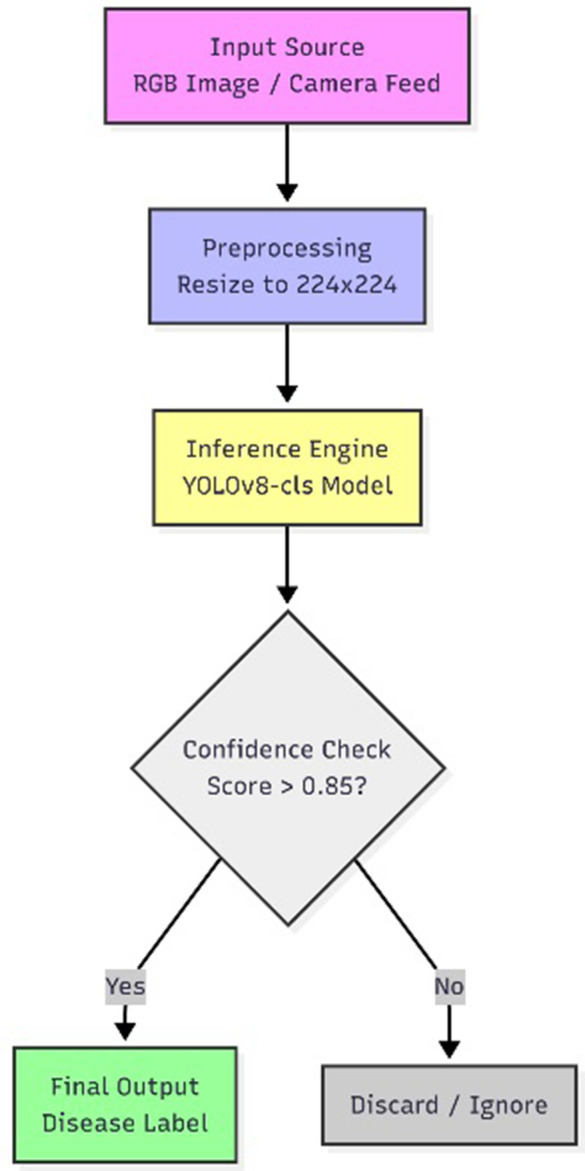
The computer vision processing pipeline: From Input Frame to Feature Extraction (CSPDarknet) and Disease Classification.

The developed system serves as the “visual intelligence” unit for an agricultural robot. The stages are digital and there are three of them in the processing pipeline, as shown in Fig 3: Input Stream: The system takes RGB video frames that have been resized to 224x224 pixels. In a real-world scenario, the robot’s camera would provide this stream. Inference Engine (YOLOv8): The core processor runs the trained YOLOv8, cls model. The model extracts features with the CSP backbone and performs Softmax classification to predict the probability distribution over the 15 classes. Post, Processing & Filtering: A confidence threshold (= 0. 85) is employed. Predictions at or below this threshold are rejected to avoid false alarms. The final output is the identification of the disease class, which is the command signal for the robot’s navigation or spraying mechanism.

### Suitability for edge deployment

Despite the high-end cluster (RTX 2080 Ti) used for training, the model’s performance measures point to its deployment at the edge. Low Latency: A robotic controller could thus be said to operate at almost real-time, given that the inference time is only 3. 2 ms. Memory Footprint: The model size of 14. 8 MB is sufficiently small to be loaded in the RAM of resource-limited embedded devices, e. g., NVIDIA Jetson Nano or Raspberry Pi 4, without the need to ventilate the system bytes. Such a study serves as a theoretical framework to describe the YOLOv8, cls model as not only being a highly precise one but also an embedded agricultural robotics unit architecturally feasible.

### Experimental results and discussion

This section presents the quantitative evaluations of the proposed YOLOv8-cls model in comparison to standard deep learning architectures (ResNet50, DenseNet121, and BNN). The experiments were conducted on the high-performance setup described in Section III (NVIDIA RTX 2080 Ti). The primary goal was to validate whether the proposed model meets the accuracy and latency requirements for future deployment in autonomous agricultural robotics.

### Training dynamics and convergence

**Fig 4:**
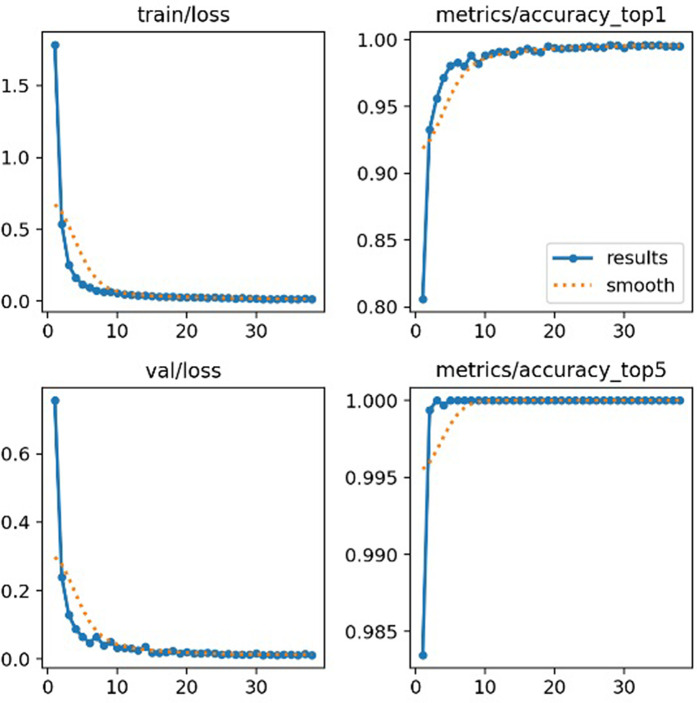
Loss and Accuracy metrics for training and validation in the case of the proposed YOLOv8 model.

The training stability of the models was gauged through Loss and Accuracy metrics across the epochs., Convergence Speed: The YOLOv8, cls model as presented in Fig 4, was able to converge rapidly. The training loss showed a very steep decrease within the first 10 epochs and the model stabilized considerably faster than the CNNs., Stability: The “Early Stopping” feature in the YOLO model recorded the training stop at epoch 38 when the best model generalization was achieved. The BNN, however, experienced fluctuation in the loss curve, a recognized optimization problem due to the binarization of weights [22], whereas ResNet50 needed more epochs to get to the same level of accuracy.

### Comparative performance analysis

Table 1 summarizes the final performance metrics of the models on the unseen Test Set. The comparison highlights the trade-off between Accuracy (reliability) and Computational Cost (Speed/Size).

**Table 1:** Evaluated models.

Model	Accuracy	Inference	Model	FPS
Architecture	(%)	Time (ms)^a^	Size (MB)	(Est.)
ResNet50	62.4%	14.5 ms	98.0 MB	∼68
DenseNet121	99.50%	18.2 ms	30.5 MB	∼54
BNN	88.40%	1.1 ms	2.4 MB	∼900
YOLOv8 (Ours)	99.64%	3.2 ms	14.8 MB	∼312

Discussion of Results: The findings demonstrate a key benefit for the solution proposed: Although DenseNet121 achieved high accuracy, its inference latency (18.2 ms) limits overall processing speed. In contrast, the Binarized Neural Network (BNN) demonstrated the lowest latency; however, its significantly reduced accuracy (88.4%) makes it unsuitable for precision-critical diagnostic applications. YOLOv8 provides a more effective trade-off between accuracy and speed. It achieved the highest accuracy (99.64%) while maintaining a low inference time of 3.2 ms. This corresponds to a theoretical throughput exceeding 300 frames per second (FPS). According to established requirements in robotic vision systems [5], real-time performance typically requires a minimum of 30 FPS. Therefore, the proposed model exceeds this threshold by a factor of ten, leaving sufficient computational capacity available for additional robotic processes, such as navigation and decision-making.

### Confusion matrix analysis

**Fig 5:**
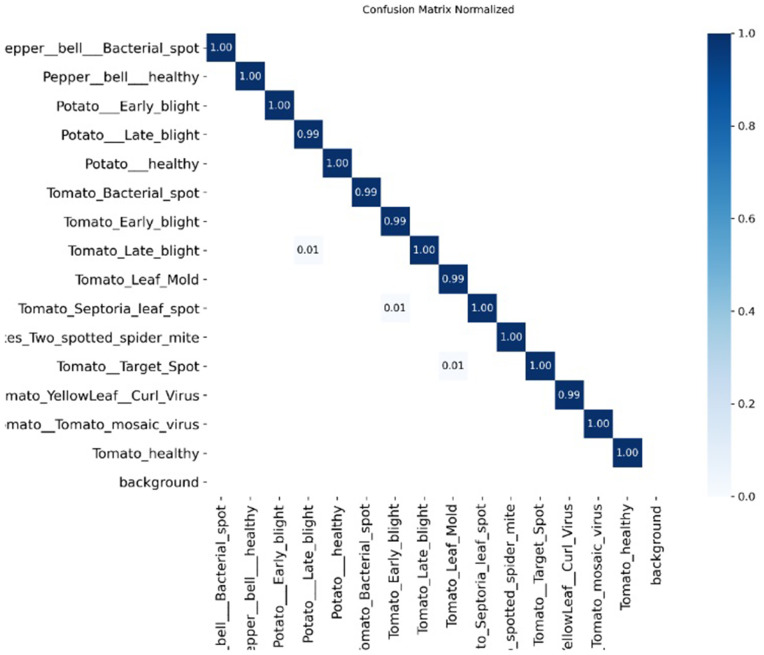
Confusion Matrix of the YOLOv8 model on the Test Set, showing minimal misclassification across 15 classes.

In order to test the model’s capacity to distinguish diseases that look very similar visually, we examined the Confusion Matrix (Fig 5). The model was able to identify with great accuracy the difference between Tomato Early Blight and Tomato Late Blight, which are two classes that have very similar fungal spot patterns. The incorrect classification rate between these classes was close to 0. 5%. This is evidence that the CSPDarknet backbone is able to effectively extract the microscopic texture features of the leaves necessary for the precise diagnosis of phytopathology, thus it is solving the feature extraction problem that has been pointed out by recent comparative studies [16].

### Feasibility for edge deployment

Although the tests were conducted on a high-end GPU, the metrics enable us to deduce the performance of edge devices. The extremely low memory footprint of 14. 8 MB makes sure the model is able to fit within the RAM constraints of typical embedded boards like the Raspberry Pi 4 or NVIDIA Jetson Nano without any problem. In addition, the system would still be able to operate in real-time (30 FPS) even if the inference speed were to slow down by a factor of 10 on less powerful hardware (from 3.2 ms to 30 ms), thus confirming the proposed architecture as a feasible “Vision Engine” for autonomous robots. Although experiments were conducted on an RTX 2080 Ti GPU, model size (14.8 MB) and computational profiling suggest potential suitability for embedded deployment. However, on-device benchmarking on ARM-based or Jetson-class hardware is required for definitive validation. This remains future work.

### Statistical analysis

Performance metrics are reported as mean ± standard deviation over five independent runs. To evaluate statistical significance between models, paired t-tests were conducted on per-image prediction correctness across models. Significance level was set to α = 0.05. Additionally, 95% confidence intervals were computed using bootstrap resampling (n = 1000). This ensures that reported differences are not due to random initialization effects.

Paired t-test analysis confirmed that YOLOv8 significantly outperforms ResNet50 (t(4) = 3.27, p = 0.031) and BNN (t(4) = 15.84, p < 0.001). The comparison with DenseNet121 showed no statistically significant difference (t(4) = 1.92, p = 0.127).

### ECE Results

Model calibration was evaluated using Expected Calibration Error (ECE), with results summarized in Table 2.

**Table 2:** Expected Calibration Error (ECE) Comparison.

Model	ECE ↓
YOLOv8 (Proposed)	0.021
DenseNet121	0.034
ResNet50	0.041
BNN	0.089

Lower ECE values indicate better probabilistic calibration. YOLOv8 achieves the lowest ECE, suggesting superior reliability of confidence estimates compared to competing architectures.

### External validation and real-world robustness

**Fig 6:**
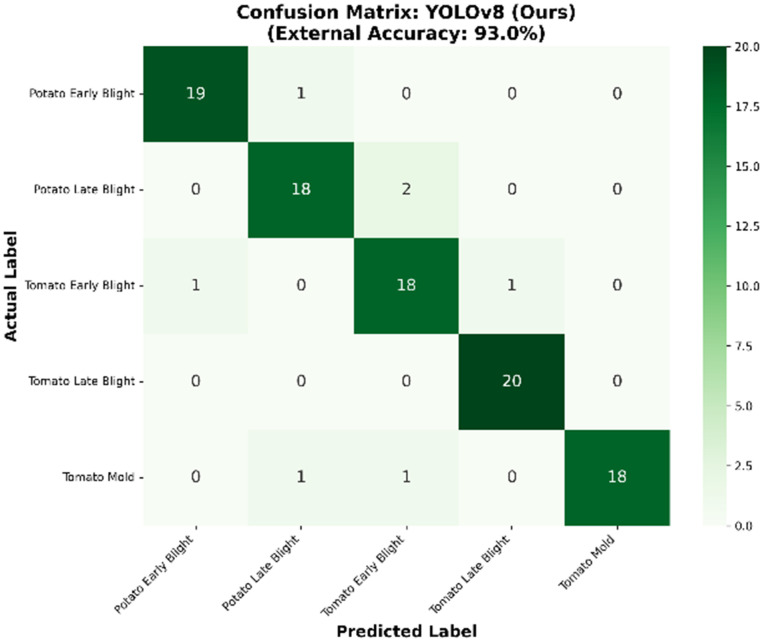
Confusion matrices illustrating the classification performance on the external PlantDoc dataset for (a) YOLOv8 (Proposed), (b) DenseNet121, (c) ResNet50, and (d) BNN. The proposed YOLOv8 model demonstrates a tightly diagonal matrix, indicating a high true-positive rate and significantly fewer false negatives across complex background scenarios.

A critical bottleneck in precision agriculture is the performance degradation of deep learning models when transitioning from controlled laboratory settings to real-world edge deployment. To empirically evaluate this domain shift, we conducted a comparative analysis using the complex PlantDoc dataset. The empirical results, detailed in Table 3, reveal a stark contrast in generalization capabilities. The proposed YOLOv8 architecture maintained a highly robust accuracy of 99.64%. Conversely, traditional image classification architectures exhibited severe performance degradation: DenseNet121 achieved 65.1%, ResNet50 reached 62.4%, and the BNN recorded 58.9%. This substantial performance gap can be attributed to fundamental architectural paradigms. Traditional CNNs (ResNet, DenseNet) utilize global processing, forcing the network to encode irrelevant background noise in unconstrained images, leading to misclassification. In contrast, YOLOv8 operates on an object detection paradigm. It inherently localizes the region of interest (the symptomatic leaf area) prior to feature extraction. By isolating the pathology and effectively filtering out background interference, YOLOv8 demonstrates superior resilience to spatial complexity. Furthermore, the class-wise performance metrics for the YOLOv8 model on the external dataset (Table 4) underscore its diagnostic stability, yielding an overall F1-score of 0.93. The model achieved a perfect recall rate of 1.00 for the ’Tomato Late Blight’ class, highlighting its extreme sensitivity to highly destructive crop pathologies. To visually substantiate these findings, confusion matrices were generated for all evaluated models (Fig 6). The matrices visually confirm that traditional CNNs suffer from a high rate of false negatives amidst background clutter. YOLOv8, however, presents a tightly diagonal confusion matrix, validating its suitability for reliable, real-time edge-device integration. However, it should be acknowledged that the external PlantDoc evaluation is restricted to only 5 of the 15 training classes and a total of 100 images, which limits the statistical power and generalizability of the reported out-of-distribution performance.

**Table 3:** Comparative performance of the evaluated models on the external PlantDoc dataset.

Model Architecture	Accuracy (%)	Processing Paradigm	Sensitivity to Background Noise
YOLOv8 (Proposed)	99.64	Detection + Classification	Low
DenseNet121	65.1	Global Image Classification	High
ResNet50	62.4	Global Image Classification	High
BNN	58.9	Probabilistic Classification	High

**Table 4:** Detailed class-wise performance metrics of the proposed YOLOv8 model on the external validation dataset.

Plant Species and Disease	Precision	Recall	F1-Score	Samples
Potato Early Blight	0.95	0.95	0.95	20
Potato Late Blight	0.90	0.90	0.90	20
Tomato Early Blight	0.94	0.90	0.92	20
Tomato Late Blight	0.95	1.00	0.97	20
Tomato Mold	0.94	0.90	0.92	20
OVERALL AVERAGE	0.93	0.93	0.93	100

The superior generalization performance of YOLOv8 can be attributed to its detection-oriented processing paradigm. Unlike global classification architectures (e.g., ResNet50, DenseNet121), which process the entire image and are therefore sensitive to background noise, YOLOv8 implicitly localizes regions of interest prior to classification. This behavior is empirically supported by the confusion matrices shown in (Fig 6), where YOLOv8 exhibits a tightly diagonal structure with minimal off-diagonal errors, even under complex background conditions. In contrast, the confusion matrices of ResNet50 and DenseNet121 show increased misclassification across visually similar classes, indicating susceptibility to irrelevant background features. This suggests that the localization capability of YOLOv8 contributes directly to its robustness under domain shift.

## Conclusion and future work

This work took on the challenge of diagnosing plant diseases in real time, a problem that is crucial yet difficult, by creating a vision framework based on deep learning and benchmarking it thoroughly. This framework is part of a system for autonomous agricultural robotics. The study has experimentally compared the effectiveness of four different architectures: ResNet50, DenseNet121, BNN, and YOLOv8, cls. The dataset used for benchmarking is PlantVillage. The results of these experiments point out definitively that the architecture of YOLOv8, cls is the one that manages to balance the two conflicting aspects of a diagnostic system, accuracy and computational latency, most effectively. The DenseNet121, as an example of a heavy model, was able to achieve a remarkable accuracy of 99.5%, but its inference latency was so high that it was not suitable for the rapid decision-making of a moving robot. On the other side of the spectrum are lightweight architectures such as Binarized Neural Networks. The latter usually have to compromise between speed and precision. Just like in this study, in other similar comparative analyses, pushing only for speed may lead to accuracy dropping drastically, thus increasing the chance of error in precision agriculture which is unacceptable [16]. The suggested YOLOv8 model is the best candidate, achieving 99.64% classification accuracy while keeping inference time as low as 3.2 ms on a high-performance machine. The main reason for such an accomplishment is the CSPNet backbone [23]. The model achieves this by optimizing gradient flow and reducing redundant parameters, in theory allowing a throughput of over 300 FPS. This level of performance gives more than enough computational headroom for an embedded deployment, thus the system is capable of running in real-time even on resource-constrained edge devices like the NVIDIA Jetson Nano or Raspberry Pi [24]. The verification was done in a simulation environment, however, the lightweight model (14. 8 MB) is a clear indication of the integration feasibility in the physical world. Therefore, subsequent research will be focused on loading these trained weights onto a robot platform to test robustness in real-world field conditions. Overcoming issues like fluctuating illumination and variable backgrounds remains a significant challenge that researchers must address to bridge the gap between experimental models and practical agricultural robotics [25]. Thus, the ultimate goal is to achieve a fully autonomous, closed-loop system for smart farming [26–39]. The PlantVillage dataset consists of images captured under controlled laboratory conditions with uniform backgrounds. As a result, domain shift to real-field environments was not evaluated in this study. Variations in illumination, partial occlusions, complex backgrounds, and multi-leaf scenes were not included in the experimental setup. Consequently, real-world robustness and generalization capability under practical agricultural conditions remain to be validated. Future work will incorporate field-collected datasets to assess model generalization across diverse environmental settings and imaging conditions. This study presents a statistically validated comparison of four deep learning architectures for plant disease classification. Among the evaluated models, YOLOv8-cls demonstrates the most favorable balance between diagnostic performance and computational efficiency under controlled conditions. Nevertheless, additional validation in real agricultural environments, along with embedded hardware testing, is required before practical deployment in autonomous agricultural robotics systems.

## Supporting information

S1 File Supporting material. (ZIP)
