# Supplementary material for: Comparative evaluation of deep learning models for plant disease classification with edge-aware performance analysis
Source: PLoS One. 2026 Jun 18;21(6):e0349901. doi: 10.1371/journal.pone.0349901 (PMC13278434; doi:10.1371/journal.pone.0349901)
Supplement: Attachment — Submitted filename: Response to Reviewers.docx [file pone.0349901.s002.docx]

Dear Editor and Reviewers,

We sincerely thank the Editor and the Reviewers for their careful evaluation of our manuscript and for the constructive and detailed comments. We have thoroughly revised the manuscript in response to all concerns raised. The revised version incorporates substantial methodological improvements, expanded statistical validation, clearer positioning of contributions, moderated claims regarding edge deployment, and extensive language corrections.

Below we provide a detailed, point-by-point response to each reviewer comment. All changes have been incorporated into the revised manuscript and highlighted accordingly.

RESPONSE TO REVIEWER #1

Comment 1: The authors have not formulated clear research questions nor used appropriate methodology.

Response:
We have now explicitly formulated four research questions in the Introduction (Section 1). Additionally, the methodology has been strengthened by:

- Implementing stratified data splitting,
- Conducting five independent experimental runs,
- Reporting mean ± standard deviation,
- Adding statistical significance testing (paired t-tests, α = 0.05),
- Including bootstrap confidence intervals (95%).

These additions ensure methodological rigor and reproducibility.

Comment 2 & 5: Lack of novelty and methodological novelty.

Response:
We have clarified the novelty in a new subsection titled “Contributions and Novelty.” The key contributions now include:

1. A statistically rigorous comparative protocol (repeated runs).
2. Formal significance testing between architectures.
3. Multi-metric evaluation beyond accuracy (macro-F1, weighted-F1, Top-2 accuracy, calibration).
4. Edge-aware performance profiling.
5. An ablation study analyzing augmentation and thresholding effects.

This distinguishes our work from standard single-run benchmarking studies.

Comment 3 & 4: Poor related work and lack of comparison with notable works.

Response:
The Related Work section has been completely reorganized into four thematic subsections:

- CNN-based classification approaches,
- Lightweight and edge-aware models,
- Detection and segmentation approaches,
- Domain generalization in plant pathology.

We have added recent high-impact journal references and clearly positioned our work relative to existing literature.

Comment 6: No statistical significance testing.

Response:
A dedicated “Statistical Analysis” section has been added. We now report:

- Mean ± standard deviation over five runs,
- Paired t-tests between models,
- 95% confidence intervals via bootstrap resampling.

These additions establish that the reported performance differences are statistically supported.

Comment 7: No ablation study.

Response:
We added an Ablation Study section evaluating:

- Effect of data augmentation,
- Confidence threshold sensitivity,
- Input resolution impact.

Results demonstrate the robustness of the selected configuration.

Comment 8: The paper does not have a clear goal.

Response:
The Introduction has been revised to clearly state the objective: to perform a statistically validated comparative evaluation of deep learning architectures for plant disease classification with emphasis on computational efficiency and edge-awareness.

RESPONSE TO REVIEWER #2

Comment: Claims regarding real-world robustness are overstated given use of PlantVillage only.

Response:
We agree. All claims regarding field robustness and robotic deployment have been moderated. A dedicated “Limitations” subsection now explicitly acknowledges:

- Controlled background bias of PlantVillage,
- Lack of domain shift evaluation,
- Absence of field validation.

Future work directions have been clearly defined.

Comment: Lack of statistical rigor.

Response:
As detailed above, we now include repeated experiments, variance reporting, and statistical testing.

Comment: Near-perfect accuracies require discussion of dataset bias.

Response:
A discussion paragraph has been added addressing:

- Controlled acquisition conditions,
- Potential overestimation of real-world performance,
- Need for cross-dataset validation.

Comment: Edge deployment conclusions are inferred from GPU results.

Response:
All deployment claims have been reframed as computational feasibility analysis rather than validated deployment. We explicitly state that on-device benchmarking (Jetson, ARM-based systems) remains future work.

Comment: Language and formatting issues.

Response:
The manuscript has undergone comprehensive language revision. Informal phrasing, typographical issues (e.g., “real, time,” “power, hungry”), and IEEE template remnants have been removed. The manuscript now follows journal-standard academic English.

RESPONSE TO REVIEWER #3

Major Comment 1: Edge/robotic deployment not empirically validated.

Response:
We have downscoped claims and clarified that this study provides an edge-aware benchmarking framework rather than a robotics validation study. Hardware extrapolation statements have been removed or qualified.

Major Comment 2: No domain generalization evaluation.

Response:
A Limitations section now explicitly addresses domain shift concerns. We acknowledge that robustness to field conditions is not demonstrated and outline plans for external validation in future work.

Major Comment 3: Experimental design lacks rigor.

Response:
We implemented:

- Stratified splitting,
- Leakage prevention checks,
- Five-run repetition protocol,
- Statistical testing.

These steps address concerns regarding reproducibility and inflated performance.

Major Comment 4: Incomplete evaluation metrics.

Response:
We now report:

- Accuracy,
- Macro and weighted precision/recall/F1,
- Top-2 accuracy,
- Calibration (ECE),
- Confusion matrices for leading models.

Latency methodology (warm-up runs, repetitions, synchronization) is also documented.

Major Comment 5: YOLOv8 architectural description inaccurate.

Response:
The YOLOv8 description has been corrected. We removed inaccurate references to CSPDarknet53 and now provide accurate architecture details consistent with Ultralytics YOLOv8-cls documentation. Full training hyperparameters are provided for reproducibility.

Major Comment 6: Robotic system not presented as full system.

Response:
We clarified that this study focuses on comparative model evaluation rather than complete robotic system implementation. System-level claims have been moderated accordingly.

Minor Comments:

- Writing and formatting corrected.
- Class distribution table added.
- Latency measurement methodology clarified.
- Prototype description expanded.
- References updated with recent journal publications.

Final Statement

We believe the revised manuscript now addresses all reviewer concerns and significantly strengthens methodological rigor, statistical validity, clarity of contribution, and transparency regarding limitations.

We sincerely appreciate the reviewers’ insightful comments, which have substantially improved the quality of this work.

Thank you for your consideration.

Sincerely,

Zirije Hasani
(on behalf of all co-authors)
