# Supplementary material for: Comparative evaluation of deep learning models for plant disease classification with edge-aware performance analysis
Source: PLoS One. 2026 Jun 18;21(6):e0349901. doi: 10.1371/journal.pone.0349901 (PMC13278434; doi:10.1371/journal.pone.0349901)
Supplement: Attachment — Submitted filename: Response_to_Reviewers_auresp_2.docx [file pone.0349901.s003.docx]

Response to Reviewers

We thank the reviewers and editor for their valuable feedback. All comments have been carefully addressed as detailed below.

# Reviewer #2

1. Duplicate Related Work section:
Resolved. The IEEE-style duplicate section has been removed, and only the PLOS-compliant version is retained.

2. ResNet50 external accuracy discrepancy:
Corrected. The consistent value of 62.4% is now used throughout the manuscript.

3. YOLOv8 accuracy discrepancy:
Corrected to 99.64% consistently across abstract and results.

4. Image dimension formatting:
All instances corrected to 224×224.

5. Track changes:
All markup removed.

6. Informal subheadings:
Replaced with neutral scientific phrasing.

7. Statistical reporting:
Exact p-values and test statistics have been added.

8. OOD limitation:
Explicitly stated in Results section.

# Recommended Improvements

Class distribution, ECE table, and extended discussion on YOLOv8 generalization have been added.

# Reviewer #3

We thank the reviewer for the positive evaluation.
