# Supplementary material for: Comparative evaluation of deep learning models for plant disease classification with edge-aware performance analysis
Source: PLoS One. 2026 Jun 18;21(6):e0349901. doi: 10.1371/journal.pone.0349901 (PMC13278434; doi:10.1371/journal.pone.0349901)
Supplement: Peer review history

# Peer review: Decision Letter 0

**DOI:** 10.1371/journal.pone.0349901.r001

**Authors:** Yile Chen

3 Feb 2026

PONE-D-26-01029

Integrating AI and robotics for visual analysis of plant health in agricultural environments

PLOS One

Dear Dr. Fondaj,

Thank you for submitting your manuscript to PLOS ONE. After careful consideration, we feel that it has merit but does not fully meet PLOS ONE’s publication criteria as it currently stands. Therefore, we invite you to submit a revised version of the manuscript that addresses the points raised during the review process.

Please submit your revised manuscript by Mar 20 2026 11:59PM. If you will need more time than this to complete your revisions, please reply to this message or contact the journal office at plosone@plos.org. When you're ready to submit your revision, log on to https://www.editorialmanager.com/pone/ and select the 'Submissions Needing Revision' folder to locate your manuscript file.

Please include the following items when submitting your revised manuscript:

- A letter that responds to each point raised by the academic editor and reviewer(s). You should upload this letter as a separate file labeled 'Response to Reviewers'.
- A marked-up copy of your manuscript that highlights changes made to the original version. You should upload this as a separate file labeled 'Revised Manuscript with Track Changes'.
- An unmarked version of your revised paper without tracked changes. You should upload this as a separate file labeled 'Manuscript'.

If you would like to make changes to your financial disclosure, please include your updated statement in your cover letter. Guidelines for resubmitting your figure files are available below the reviewer comments at the end of this letter.

If applicable, we recommend that you deposit your laboratory protocols in protocols.io to enhance the reproducibility of your results. Protocols.io assigns your protocol its own identifier (DOI) so that it can be cited independently in the future. For instructions see: https://journals.plos.org/plosone/s/submission-guidelines#loc-laboratory-protocols. Additionally, PLOS ONE offers an option for publishing peer-reviewed Lab Protocol articles, which describe protocols hosted on protocols.io. Read more information on sharing protocols at https://plos.org/protocols?utm_medium=editorial-email&utm_source=authorletters&utm_campaign=protocols.

We look forward to receiving your revised manuscript.

Kind regards,

Yile Chen, Ph.D. in Architecture

Academic Editor

PLOS One

Journal Requirements:

When submitting your revision, we need you to address these additional requirements.

1. Please ensure that your manuscript meets PLOS ONE's style requirements, including those for file naming. The PLOS ONE style templates can be found at

https://journals.plos.org/plosone/s/file?id=wjVg/PLOSOne_formatting_sample_main_body.pdf and

https://journals.plos.org/plosone/s/file?id=ba62/PLOSOne_formatting_sample_title_authors_affiliations.pdf

2. Please note that PLOS One has specific guidelines on code sharing for submissions in which author-generated code underpins the findings in the manuscript. In these cases, we expect all author-generated code to be made available without restrictions upon publication of the work. Please review our guidelines at https://journals.plos.org/plosone/s/materials-and-software-sharing#loc-sharing-code and ensure that your code is shared in a way that follows best practice and facilitates reproducibility and reuse.

3. Please update your submission to use the PLOS LaTeX template. The template and more information on our requirements for LaTeX submissions can be found at http://journals.plos.org/plosone/s/latex.

4. We note that your Data Availability Statement is currently as follows: “All relevant data are within the manuscript and its Supporting Information files.”

Please confirm at this time whether or not your submission contains all raw data required to replicate the results of your study. Authors must share the “minimal data set” for their submission. PLOS defines the minimal data set to consist of the data required to replicate all study findings reported in the article, as well as related metadata and methods (https://journals.plos.org/plosone/s/data-availability#loc-minimal-data-set-definition).

For example, authors should submit the following data:

- The values behind the means, standard deviations and other measures reported;

- The values used to build graphs;

- The points extracted from images for analysis.

Authors do not need to submit their entire data set if only a portion of the data was used in the reported study.

If your submission does not contain these data, please either upload them as Supporting Information files or deposit them to a stable, public repository and provide us with the relevant URLs, DOIs, or accession numbers. For a list of recommended repositories, please see https://journals.plos.org/plosone/s/recommended-repositories.

If there are ethical or legal restrictions on sharing a de-identified data set, please explain them in detail (e.g., data contain potentially sensitive information, data are owned by a third-party organization, etc.) and who has imposed them (e.g., an ethics committee). Please also provide contact information for a data access committee, ethics committee, or other institutional body to which data requests may be sent. If data are owned by a third party, please indicate how others may request data access.

5. PLOS requires an ORCID iD for the corresponding author in Editorial Manager on papers submitted after December 6th, 2016. Please ensure that you have an ORCID iD and that it is validated in Editorial Manager. To do this, go to ‘Update my Information’ (in the upper left-hand corner of the main menu), and click on the Fetch/Validate link next to the ORCID field. This will take you to the ORCID site and allow you to create a new iD or authenticate a pre-existing iD in Editorial Manager.

6. Please ensure that you refer to Figures 1 and 2 in your text as, if accepted, production will need this reference to link the reader to the figure.

7. If the reviewer comments include a recommendation to cite specific previously published works, please review and evaluate these publications to determine whether they are relevant and should be cited. There is no requirement to cite these works unless the editor has indicated otherwise.

[Note: HTML markup is below. Please do not edit.]

Reviewers' comments:

Reviewer's Responses to Questions

**Comments to the Author**

1. Is the manuscript technically sound, and do the data support the conclusions?

The manuscript must describe a technically sound piece of scientific research with data that supports the conclusions. Experiments must have been conducted rigorously, with appropriate controls, replication, and sample sizes. The conclusions must be drawn appropriately based on the data presented.

Reviewer #1: No

Reviewer #2: Partly

Reviewer #3: Yes

**********

2. Has the statistical analysis been performed appropriately and rigorously?

Reviewer #1: No

Reviewer #2: No

Reviewer #3: Yes

**********

3. Have the authors made all data underlying the findings in their manuscript fully available?

The PLOS Data policy requires authors to make all data underlying the findings described in their manuscript fully available without restriction, with rare exception (please refer to the Data Availability Statement in the manuscript PDF file). The data should be provided as part of the manuscript or its supporting information, or deposited to a public repository. For example, in addition to summary statistics, the data points behind means, medians and variance measures should be available. If there are restrictions on publicly sharing data—e.g. participant privacy or use of data from a third party—those must be specified.

Reviewer #1: Yes

Reviewer #2: Yes

Reviewer #3: Yes

**********

4. Is the manuscript presented in an intelligible fashion and written in standard English?

PLOS ONE does not copyedit accepted manuscripts, so the language in submitted articles must be clear, correct, and unambiguous. Any typographical or grammatical errors should be corrected at revision, so please note any specific errors here.

Reviewer #1: No

Reviewer #2: Yes

Reviewer #3: Yes

**********

5. Review Comments to the Author

Please use the space provided to explain your answers to the questions above. You may also include additional comments for the author, including concerns about dual publication, research ethics, or publication ethics. (Please upload your review as an attachment if it exceeds 20,000 characters)

Reviewer #1: The following are my comments:

1. Authors have not really formulated research questions and use appropriate methodology to find answers to the research questions framed.

2. I do not find much novelty in this work. Authors should make it clear what is novel in this work.

3. There is no comparison with notable works in this area.

4. Related work section is extremely poor.

5. There is no methodological novelty.

6. No statistical significance test was conducted to establish that the proposed work is significantly better.

7. No ablation study was conducted.

8. The paper does not have a very clear goal.

Reviewer #2: The manuscript addresses an important problem in precision agriculture and presents a comparative evaluation of deep learning models for plant disease classification with an emphasis on computational efficiency. The implementation is technically sound, and the experimental results suggest that YOLOv8 provides a favorable trade-off between accuracy and inference latency.

However, several issues should be addressed to strengthen the manuscript. First, all experiments are conducted exclusively on the PlantVillage dataset, which is captured under controlled conditions. As a result, claims regarding real-world robustness, field deployment, and robotic applicability should be moderated or explicitly qualified. Second, the comparative analysis lacks statistical rigor, as results are reported only as single point estimates without confidence intervals, variance, or significance testing. This limits the strength of the performance comparisons, particularly given the marginal accuracy differences between models. Third, the reported near-perfect accuracies (>99%) warrant discussion of potential dataset bias and generalization limitations. Finally, conclusions regarding edge deployment are based on inference from GPU-based experiments rather than direct evaluation on embedded hardware, which should be acknowledged as a limitation.

In addition, the manuscript requires substantial language and formatting revision to meet journal standards, as it contains numerous grammatical errors and stylistic inconsistencies.

Reviewer #3: 1) Summary and overall assessment

The manuscript presents a comparative evaluation of four deep learning approaches (ResNet50, DenseNet121, a binarized neural network, and YOLOv8-cls) on the PlantVillage dataset for leaf disease classification, with an application framing toward robotic and edge deployment. The authors report that YOLOv8-cls achieves the best trade-off among accuracy, inference latency, and model size (reported accuracy 99.64%, latency 3.2 ms, model size 14.8 MB), and they mention a web-based prototype as a proof-of-concept.

The topic is relevant to precision agriculture and embedded vision. However, in its current form, the work reads as a lightweight benchmark on a laboratory dataset rather than a robotics-validated study, and several methodological and reporting gaps prevent the results from being considered robust, reproducible, and representative of real agricultural conditions. Substantial revision is required before the contribution can meet the standards expected in a high-impact venue.

2) Major comments (must address)

Major Comment 1 — Robotics/edge deployment claims are not empirically substantiated

Although the paper positions the contribution as “robotic and edge deployment” focused, all timing results are reported from a high-end GPU environment (RTX 2080 Ti). The manuscript then extrapolates feasibility to edge devices by assuming a slowdown factor and inferring that real-time performance would still hold. This is not sufficient for a deployment-oriented claim.

Required improvements

Provide on-device benchmarking on at least one realistic edge platform (e.g., Raspberry Pi 4/5, Jetson Nano/Orin Nano, Coral TPU, or a common ARM SoC) with:

end-to-end latency (capture → preprocess → inference → postprocess),

throughput (FPS) under realistic camera stream settings,

memory usage (peak RAM),

power/energy (W, J/frame) if possible.

Specify inference settings (batch size, precision FP32/FP16/INT8, TorchScript/ONNX/TensorRT use, CPU/GPU utilization).

If hardware testing is not feasible, then the manuscript must re-scope the claims: frame the work as a dataset-level model comparison rather than an edge robotics validation.

Major Comment 2 — PlantVillage does not represent field conditions; generalization is not demonstrated

PlantVillage is widely known to be a controlled dataset (often clean backgrounds, constrained acquisition), and high accuracies can be obtained without reflecting real-world robustness. The manuscript does not evaluate domain shift (field backgrounds, illumination changes, occlusions, motion blur, multiple leaves per frame) which are central to robotic agricultural environments.

Required improvements

Add at least one external validation dataset or field-captured test set (even modest in size) to quantify domain generalization.

Alternatively, apply domain shift protocols:

Train on PlantVillage, test on a “real field” dataset (or vice versa),

Report accuracy drop and analyze failure modes.

Provide robustness tests: varying lighting, blur, compression, partial occlusion, and cluttered backgrounds—ideally with controlled perturbation experiments and ablations.

Major Comment 3 — Experimental design lacks rigor: splitting strategy, leakage control, and statistical confidence

The paper reports a single 70/15/15 split and single-point metrics. There is no evidence that the split is stratified by class or that near-duplicate images are controlled. PlantVillage may contain visually similar samples; without robust splitting, inflated performance is possible.

Required improvements

Use stratified splitting and report per-class sample counts in each partition.

Consider repeated trials (e.g., 5 runs with different random seeds) or k-fold cross-validation; report mean ± std (or confidence intervals).

Explicitly document leakage prevention steps (duplicate detection, ensuring no near-identical samples appear across splits).

Provide statistical comparisons (e.g., paired tests on per-image correctness across models, or bootstrap CIs for key metrics).

Major Comment 4 — Evaluation metrics are incomplete for a diagnostic setting

Accuracy alone is insufficient, especially for multi-class disease classification where class imbalance and clinically relevant errors matter. The confusion matrix is discussed for YOLOv8, but equivalent diagnostic detail is not provided for other models.

Required improvements

Report macro and weighted precision, recall, F1-score; provide per-class metrics.

Provide confusion matrices for all models or at minimum for the two top performers.

Include calibration assessment (e.g., reliability diagram/ECE) if the system is meant to drive robotic actions (spraying, navigation). A confidence threshold is used, but its effect is not quantified.

Report Top-2 or Top-3 accuracy (common in multi-class classification) and justify operational decision rules.

Major Comment 5 — Model descriptions and technical details are partially inaccurate or underspecified

The manuscript attributes a “CSPDarknet53 backbone” to YOLOv8 classification. YOLOv8’s architecture details differ from earlier YOLO generations; classification variants also differ from detection models. The paper should be technically precise and give reproducible details.

Required improvements

Correctly describe the YOLOv8-cls architecture used (variant name, depth/width scaling, input resolution, augmentation pipeline, optimizer settings).

Provide complete training hyperparameters for each model:

epochs, batch size, learning rate schedule, weight decay, augmentation parameters,

early stopping criterion implementation details,

initialization / pretrained weights used (and sources).

Clearly define the BNN architecture (layer types, binarization method, training trick such as STE, optimizer, and any scaling factors). Currently it is described conceptually but not reproducibly.

Major Comment 6 — The “robotic system” is not actually presented as a system contribution

The paper mentions an agricultural robot pipeline and a web-based prototype, but provides no system architecture diagram with implementation-level details, no robotics integration results, and no user/system evaluation.

Required improvements

If robotics integration is a central contribution, include:

system design (sensors, compute board, camera specs),

perception-to-action loop description (how classification triggers decisions),

demonstration scenario(s) and qualitative results,

failure handling and safety logic (false positives/negatives consequences).

If not, downscope: position as a comparative model study with a web demo, and remove strong robotics claims.

3) Minor comments (should address)

Writing quality and formatting: The manuscript contains multiple typographical and style issues (e.g., pervasive “real, time”, “power, hungry”, “error, prone”), and remnants of an IEEE template header (“XXX-X-XXXX… ©20XX IEEE”). These will be unacceptable in a journal submission and should be thoroughly corrected.

PONE-D-26-01029_reviewer

Clarity of contribution: The contribution list in the introduction overlaps with what is standard benchmarking. It should be reframed as explicit novelty: e.g., edge-aware benchmarking protocol, robustness evaluation, quantization study, or robotic validation.

Dataset reporting: The paper states “15 classes” but does not provide the exact class list and sample counts per class. Include a table.

Fairness of comparisons: Ensure all models are trained under comparable conditions (same input size, augmentation policy, and comparable optimization budgets). If YOLOv8 uses different defaults from Ultralytics training, that advantage must be controlled or explicitly justified.

Latency methodology: Report latency measurement method (warm-up runs, number of repetitions, single-image vs batched, CPU/GPU synchronization). Without this, ms-level comparisons are not reliable.

Prototype description: A “web-based prototype” is stated but not described (stack, inference location, model served how, response time). Include screenshots and minimal performance metrics.

References: Several citations are conference proceedings and may be acceptable, but the related work should include more recent, high-quality journal studies on domain generalization and field deployment in plant pathology imaging, and differentiate classification vs detection/segmentation tasks.

**********

6. PLOS authors have the option to publish the peer review history of their article (what does this mean?). If published, this will include your full peer review and any attached files.

If you choose “no”, your identity will remain anonymous but your review may still be made public.

**Do you want your identity to be public for this peer review?**   For information about this choice, including consent withdrawal, please see our Privacy Policy.

Reviewer #1: No

Reviewer #2: No

Reviewer #3: **Yes:**   Rapeepan Pitakaso

**********

[NOTE: If reviewer comments were submitted as an attachment file, they will be attached to this email and accessible via the submission site. Please log into your account, locate the manuscript record, and check for the action link "View Attachments". If this link does not appear, there are no attachment files.]

To ensure your figures meet our technical requirements, please review our figure guidelines: https://journals.plos.org/plosone/s/figures

You may also use PLOS’s free figure tool, NAAS, to help you prepare publication quality figures: https://journals.plos.org/plosone/s/figures#loc-tools-for-figure-preparation.

NAAS will assess whether your figures meet our technical requirements by comparing each figure against our figure specifications.

# Author comment: Author response to Decision Letter 1

**DOI:** 10.1371/journal.pone.0349901.r002

1 Apr 2026

Dear Editor,

Based on your letter, I confirm that both accounts belong to me. Please merge them in order to resolve the problem with my ORCID iD.

Kind regards,

Attachment Submitted filename: Response to Reviewers.docx

# Peer review: Decision Letter 1

**DOI:** 10.1371/journal.pone.0349901.r003

**Authors:** Yile Chen

28 Apr 2026

PONE-D-26-01029R1

Comparative Evaluation of Deep Learning Models for Plant Disease Classification with Edge-Aware Performance Analysis

PLOS One

Dear Dr. Fondaj,

Thank you for submitting your manuscript to PLOS ONE. After careful consideration, we feel that it has merit but does not fully meet PLOS ONE’s publication criteria as it currently stands. Therefore, we invite you to submit a revised version of the manuscript that addresses the points raised during the review process.

Please submit your revised manuscript by Jun 12 2026 11:59PM. If you will need more time than this to complete your revisions, please reply to this message or contact the journal office at plosone@plos.org. When you're ready to submit your revision, log on to https://www.editorialmanager.com/pone/ and select the 'Submissions Needing Revision' folder to locate your manuscript file.

Please include the following items when submitting your revised manuscript:

- A letter that responds to each point raised by the academic editor and reviewer(s). You should upload this letter as a separate file labeled 'Response to Reviewers'.
- A marked-up copy of your manuscript that highlights changes made to the original version. You should upload this as a separate file labeled 'Revised Manuscript with Track Changes'.
- An unmarked version of your revised paper without tracked changes. You should upload this as a separate file labeled 'Manuscript'.

If you would like to make changes to your financial disclosure, please include your updated statement in your cover letter. Guidelines for resubmitting your figure files are available below the reviewer comments at the end of this letter.

If applicable, we recommend that you deposit your laboratory protocols in protocols.io to enhance the reproducibility of your results. Protocols.io assigns your protocol its own identifier (DOI) so that it can be cited independently in the future. For instructions see: https://journals.plos.org/plosone/s/submission-guidelines#loc-laboratory-protocols. Additionally, PLOS ONE offers an option for publishing peer-reviewed Lab Protocol articles, which describe protocols hosted on protocols.io. Read more information on sharing protocols at https://plos.org/protocols?utm_medium=editorial-email&utm_source=authorletters&utm_campaign=protocols.

As the corresponding author, your ORCID iD is verified in the submission system and will appear in the published article. PLOS supports the use of ORCID, and we encourage all coauthors to register for an ORCID iD and use it as well. Please encourage your coauthors to verify their ORCID iD within the submission system before final acceptance, as unverified ORCID iDs will not appear in the published article. *Only*   the individual author can complete the verification step; PLOS staff *cannot*   verify ORCID iDs on behalf of authors.

We look forward to receiving your revised manuscript.

Kind regards,

Yile Chen, Ph.D. in Architecture

Academic Editor

PLOS One

Journal Requirements:

If the reviewer comments include a recommendation to cite specific previously published works, please review and evaluate these publications to determine whether they are relevant and should be cited. There is no requirement to cite these works unless the editor has indicated otherwise.

Please review your reference list to ensure that it is complete and correct. If you have cited papers that have been retracted, please include the rationale for doing so in the manuscript text, or remove these references and replace them with relevant current references. Any changes to the reference list should be mentioned in the rebuttal letter that accompanies your revised manuscript. If you need to cite a retracted article, indicate the article’s retracted status in the References list and also include a citation and full reference for the retraction notice.

**Additional Editor Comments:**

There are still some issues with the wording that require further clarification and modification.

[Note: HTML markup is below. Please do not edit.]

Reviewers' comments:

Reviewer's Responses to Questions

**Comments to the Author**

1. If the authors have adequately addressed your comments raised in a previous round of review and you feel that this manuscript is now acceptable for publication, you may indicate that here to bypass the “Comments to the Author” section, enter your conflict of interest statement in the “Confidential to Editor” section, and submit your "Accept" recommendation.

Reviewer #2: All comments have been addressed

Reviewer #3: All comments have been addressed

**********

2. Is the manuscript technically sound, and do the data support the conclusions?

The manuscript must describe a technically sound piece of scientific research with data that supports the conclusions. Experiments must have been conducted rigorously, with appropriate controls, replication, and sample sizes. The conclusions must be drawn appropriately based on the data presented.

Reviewer #2: Partly

Reviewer #3: Yes

**********

3. Has the statistical analysis been performed appropriately and rigorously?

Reviewer #2: Yes

Reviewer #3: Yes

**********

4. Have the authors made all data underlying the findings in their manuscript fully available?

The PLOS Data policy requires authors to make all data underlying the findings described in their manuscript fully available without restriction, with rare exception (please refer to the Data Availability Statement in the manuscript PDF file). The data should be provided as part of the manuscript or its supporting information, or deposited to a public repository. For example, in addition to summary statistics, the data points behind means, medians and variance measures should be available. If there are restrictions on publicly sharing data—e.g. participant privacy or use of data from a third party—those must be specified.

Reviewer #2: Yes

Reviewer #3: Yes

**********

5. Is the manuscript presented in an intelligible fashion and written in standard English?

PLOS ONE does not copyedit accepted manuscripts, so the language in submitted articles must be clear, correct, and unambiguous. Any typographical or grammatical errors should be corrected at revision, so please note any specific errors here.

Reviewer #2: Yes

Reviewer #3: Yes

**********

6. Review Comments to the Author

Please use the space provided to explain your answers to the questions above. You may also include additional comments for the author, including concerns about dual publication, research ethics, or publication ethics. (Please upload your review as an attachment if it exceeds 20,000 characters)

Reviewer #2: Thank you for your thorough revision. The revisions address the major concerns raised in the previous round, and the methodological improvements are genuine. However, several issues must be resolved before this manuscript can be accepted.

Required corrections before acceptance:

- Resolve the duplicate Related Work section. The manuscript currently contains two versions of this section with incompatible citation numbering. The PLOS-format version should be retained and the IEEE-format version removed.

- Clarify the ResNet50 external accuracy. The two versions of Table 2 (the PLOS version and the IEEE version included in the submission) report 62.4% and 42.4% respectively. The correct value must be stated clearly and used consistently.

- Resolve the abstract accuracy discrepancy for YOLOv8 (99.61% vs. 99.64%).

- Fix the formatting of image dimensions throughout ("224 224" should be "224×224").

- Remove all remaining track-change markup from the submitted manuscript.

- Replace informal subheadings in the results discussion ("The BNN Failure," "The Optimal Balance") with neutral descriptive headings or incorporate the content into running prose.

- Report the exact p-values and test statistics from the paired t-tests.

- Add a sentence in the results section (not only the conclusion) explicitly noting that the PlantDoc OOD evaluation covers only 5 of the 15 training classes and 100 images, and that this limits the strength of generalization claims.

Recommended improvements (not mandatory for acceptance):

- Provide the class distribution of the 15-class PlantVillage subset used (20,639 images) to allow readers to assess class balance independently of the authors' claims.

- Consider reporting Expected Calibration Error (ECE) values numerically in a table rather than mentioning their computation without presenting the results.

- Strengthen the discussion of why YOLOv8's object detection paradigm confers a generalization advantage over global classification architectures. The localization explanation offered is plausible but would benefit from reference to the confusion matrix evidence.

Reviewer #3: Well done, the authors has executed every required comment and make the article muxh better and easier to read

**********

7. PLOS authors have the option to publish the peer review history of their article (what does this mean?). If published, this will include your full peer review and any attached files.

If you choose “no”, your identity will remain anonymous but your review may still be made public.

**Do you want your identity to be public for this peer review?**   For information about this choice, including consent withdrawal, please see our Privacy Policy.

Reviewer #2: No

Reviewer #3: **Yes:**   Rapeepan pitakaso

**********

[NOTE: If reviewer comments were submitted as an attachment file, they will be attached to this email and accessible via the submission site. Please log into your account, locate the manuscript record, and check for the action link "View Attachments". If this link does not appear, there are no attachment files.]

To ensure your figures meet our technical requirements, please review our figure guidelines: https://journals.plos.org/plosone/s/figures

You may also use PLOS’s free figure tool, NAAS, to help you prepare publication quality figures: https://journals.plos.org/plosone/s/figures#loc-tools-for-figure-preparation.

NAAS will assess whether your figures meet our technical requirements by comparing each figure against our figure specifications.

# Author comment: Author response to Decision Letter 2

**DOI:** 10.1371/journal.pone.0349901.r004

5 May 2026

Response to Reviewers

We thank the reviewers and editor for their valuable feedback. All comments have been carefully addressed as detailed below.

Reviewer #2

1. Duplicate Related Work section:

Resolved. The IEEE-style duplicate section has been removed, and only the PLOS-compliant version is retained.

2. ResNet50 external accuracy discrepancy:

Corrected. The consistent value of 62.4% is now used throughout the manuscript.

3. YOLOv8 accuracy discrepancy:

Corrected to 99.64% consistently across abstract and results.

4. Image dimension formatting:

All instances corrected to 224×224.

5. Track changes:

All markup removed.

6. Informal subheadings:

Replaced with neutral scientific phrasing.

7. Statistical reporting:

Exact p-values and test statistics have been added.

8. OOD limitation:

Explicitly stated in Results section.

Recommended Improvements

Class distribution, ECE table, and extended discussion on YOLOv8 generalization have been added.

Reviewer #3

We thank the reviewer for the positive evaluation.

Attachment Submitted filename: Response_to_Reviewers_auresp_2.docx

# Editor report: Decision Letter 2

**DOI:** 10.1371/journal.pone.0349901.r005

**Authors:** Yile Chen

7 May 2026

Comparative Evaluation of Deep Learning Models for Plant Disease Classification with Edge-Aware Performance Analysis

PONE-D-26-01029R2

Dear Dr. Jakup,

We’re pleased to inform you that your manuscript has been judged scientifically suitable for publication and will be formally accepted for publication once it meets all outstanding technical requirements.

Within one week, you’ll receive an e-mail detailing the required amendments. When these have been addressed, you’ll receive a formal acceptance letter and your manuscript will be scheduled for publication.

An invoice will be generated when your article is formally accepted. Please note, if your institution has a publishing partnership with PLOS and your article meets the relevant criteria, all or part of your publication costs will be covered. Please make sure your user information is up-to-date by logging into Editorial Manager at Editorial Manager® and clicking the ‘Update My Information' link at the top of the page. For questions related to billing, please contact billing support.

If your institution or institutions have a press office, please notify them about your upcoming paper to help maximize its impact. If they’ll be preparing press materials, please inform our press team as soon as possible -- no later than 48 hours after receiving the formal acceptance. Your manuscript will remain under strict press embargo until 2 pm Eastern Time on the date of publication. For more information, please contact onepress@plos.org.

Kind regards,

Yile Chen, Ph.D. in Architecture

Academic Editor

PLOS One

Additional Editor Comments (optional):

The previous reviewers indicated that the manuscript was acceptable. Only a few minor spelling and grammatical errors needed correction, which the authors have already made.

Reviewers' comments:

# Editor report: Acceptance letter

**DOI:** 10.1371/journal.pone.0349901.r006

**Authors:** Yile Chen

PONE-D-26-01029R2

PLOS One

Dear Dr. Fondaj,

I'm pleased to inform you that your manuscript has been deemed suitable for publication in PLOS One. Congratulations! Your manuscript is now being handed over to our production team.

At this stage, our production department will prepare your paper for publication. This includes ensuring the following:

* All references, tables, and figures are properly cited

* All relevant supporting information is included in the manuscript submission,

* There are no issues that prevent the paper from being properly typeset

You will receive further instructions from the production team, including instructions on how to review your proof when it is ready. Please keep in mind that we are working through a large volume of accepted articles, so please give us a few days to review your paper and let you know the next and final steps.

Lastly, if your institution or institutions have a press office, please let them know about your upcoming paper now to help maximize its impact. If they'll be preparing press materials, please inform our press team within the next 48 hours. Your manuscript will remain under strict press embargo until 2 pm Eastern Time on the date of publication. For more information, please contact onepress@plos.org.

You will receive an invoice from PLOS for your publication fee after your manuscript has reached the completed accept phase. If you receive an email requesting payment before acceptance or for any other service, this may be a phishing scheme. Learn how to identify phishing emails and protect your accounts at https://explore.plos.org/phishing.

If we can help with anything else, please email us at customercare@plos.org.

Thank you for submitting your work to PLOS ONE and supporting open access.

Kind regards,

PLOS ONE Editorial Office Staff

on behalf of

Dr. Yile Chen

Academic Editor

PLOS One
